# Antimicrobial and Anti-Proliferative Effects of Skin Mucus Derived from *Dasyatis pastinaca* (Linnaeus, 1758)

**DOI:** 10.3390/md15110342

**Published:** 2017-11-01

**Authors:** Virginia Fuochi, Giovanni Li Volti, Giuseppina Camiolo, Francesco Tiralongo, Cesarina Giallongo, Alfio Distefano, Giulio Petronio Petronio, Ignazio Barbagallo, Maria Viola, Pio Maria Furneri, Michelino Di Rosa, Roberto Avola, Daniele Tibullo

**Affiliations:** 1 Department of Biomedical and Biotechnological Sciences, University of Catania, Catania 95124, Italy; vfuochi@unict.it (V.F.); livolti@unict.it (G.L.V.); giusicamiolo.89@gmail.com (G.C.); cesarinagiallongo@yahoo.it (C.G.); distalfio@gmail.com (A.D.); gpetroniopetronio@gmail.com (G.P.P.); mviola@unict.it (M.V.); furneri@unict.it (P.M.F.); chitotriosidase@gmail.com (M.D.R.); ravola@unict.it (R.A.); 2 Ente Fauna Marina Mediterranea, Avola, Siracusa 96012, Italy; fra.tiralongo@hotmail.it; 3 Department of Drug Sciences, University of Catania, Catania 95125, Italy; ignazio.barbagallo@unict.it

**Keywords:** antimicrobial, fish mucus, cytotoxicity, chitinase, Pastinaca

## Abstract

Resistance to chemotherapy occurs in various diseases (i.e., cancer and infection), and for this reason, both are very difficult to treat. Therefore, novel antimicrobial and chemotherapic drugs are needed for effective antibiotic therapy. The aim of the present study was to assess the antimicrobial and anti-proliferative effects of skin mucus derived from *Dasyatis pastinaca* (Linnaeus, 1758). Our results showed that skin mucus exhibited a significant and specific antibacterial activity against Gram-negative bacteria but not against Gram-positive bacteria. Furthermore, we also observed a significant antifungal activity against some strains of *Candida* spp. Concerning anti-proliferative activity, we showed that fish mucus was specifically toxic for acute leukemia cells (HL60) with an inhibition of proliferation in a dose dependent manner (about 52% at 1000 μg/mL of fish skin mucous, FSM). Moreover, we did not observe effects in healthy cells, in neuroblastoma cells (SH-SY5Y), and multiple myeloma cell lines (MM1, U266). Finally, it exhibited strong expression and activity of chitinase which may be responsible, at least in part, for the aforementioned results.

## 1. Introduction

The rise and spread of antibiotic resistance represent a unique challenge to both science and medicine. Resistance to chemotherapy evolves in only two types of diseases—cancer and infection, and for this reason, both are very difficult to treat [1]. Therefore, several studies were recently carried out in order to identify new antimicrobial agents to overcome such issue. Interestingly, several natural agents have been shown to exhibit various grade of antimicrobial activity and among them, fish mucus is emerging as one of the most active. The external body of fish is covered by a layer of mucus secreted by specialized cells in the epidermal layer conferring mechanical protective and lubricant functions as well as reducing body friction in water and thus assisting in swimming [2,3]. In addition, it also contains a variety of biologically active substances, such as complements, immunoglobulins, lectins, protease inhibitors, and lytic enzymes—including lysozyme—serving as defense substances [2,3]. In fact, the aquatic environment contains different types of pathogens and the mucus layer provides a physical barrier acting as a first line of defense playing an important role in the innate immunity of fish. To this regard, Austin et al. demonstrated that the mucus extract from the surface of rainbow trout (*Salmo gairdneri*) exhibited antibacterial activity [4]. Similarly, other authors identified a unique antibacterial spectrum of the mucus in rabbit fishes and partially characterized the antibacterial factor of the *S. fuscescens* [5]. Similarly, Hellio et al. performed a screening for antifungal, antibacterial, and cytotoxic activities of epidermal mucus and epidermis extracts of thirteen fish species [3,6,7,8]. Consistently, the antimicrobial property of crude skin mucus against microbial pathogens was also demonstrated for other fishes such as *Rita rita* and *Channa punctatus* [3]. As far as concern putative molecules responsible for such activities, previous reports suggested that fish skin mucus possesses a variety of biologically active substances such as lectins, proteolytic enzymes, flavoenzymes, protein, apolipoprotein A–I, and antimicrobial peptides that are constitutively expressed to provide immediate protection to fish from potential pathogens but also antitumor activity [9,10,11]. In addition, Ben et al. showed the antibacterial effects of intestinal phospholipase A2 (stingray phospholipase A2 group IIA, sPLA 2-IIA) from the common stingray *D. pastinaca* [12,13]. Interestingly, several studies showed that skin mucus has a cytotoxic effect also in human cancer cells [14]. 

The aim of the present study was to evaluate the bioactive properties of crude skin mucus derived from *Dasyatis pastinaca* with particular regard to the antiproliferative effects in healthy and cancerous cells and its antimicrobial effect. This species, together with other elasmobranchs, is not commercially valuable and is usually discarded by fishermen and therefore developing new products from FSM may transform a wasted product into a significant source.

## 2. Materials and Methods

### 2.1. Growth Inhibition Curves by Cell Counting

The growth inhibition activity was tested for the following pathogens: *Escherichia coli* ATCC 25922, *E. coli* ATCC 35218, *Enterococcus faecalis* ATCC 29212, *Staphylococcus aureus* ATCC 25923, *Pseudomonas aeruginosa* ATCC 27853, *Klebsiella pneumoniae* ATCC 700603, and *Streptococcus agalactiae* DSM 2134. A bacterial suspension of 0.5 McFarland (1.5 × 10^8^ CFU/mL) was prepared for each pathogen, after an overnight subculture in Mueller-Hinton broth (MH broth), measuring OD at 600 nm (Model 680 Microplate Reader, Bio-Rad, Milan, Italy). A series of dilutions were prepared in order to obtain a final concentration of 7.5 × 10^5^ CFU/mL in MH broth. The fish skin mucous (FSM) was added in a concentration range from 16.50 µg/µL to 0.06 µg/µL. Inoculated bottles were incubated aerobically with shaking at 37 °C; 100 µL of each dilution were taken every 60 min for 22 h for cell counting. For this purpose, serial 10-fold dilutions in sterile saline (NaCl 0.85% *w*/*v*) were performed, and each dilution was spread on MH agar [15]. All measurements were repeated six times. 

### 2.2. Antifungal Assay

A suspension of overnight culture of each strain of *Candida* spp. (*Candida albicans* ATCC90028, *Candida albicans* ATCC10231, *Candida albicans* clinical strain 6, *Candida albicans* clinical strain 10, *Candida glabrata* clinical strain 14, *Candida tropicalis* clinical strain 21) grown on Sabouraud chloramphenicol agar plates (SAB Dex Chlor) was prepared in sterile saline (NaCl 0.85% *w*/*v*). Suspensions were adjusted to 1.0 × 10^6^ CFU/mL (0.5 McFarland) and then diluted to obtain a concentration of 1.0 × 10^4^ CFU/mL in RPMI 1640.

The activity of the extract against *Candida* strains was determined by microplate assay following the Clinical and Laboratory Standards Institute document M27-A3 [16]. A mixture of 100 µL *C. albicans* suspensions in RPMI broth (final concentration 0.5 × 10^3^ CFU/mL) and 100 µL of FSM were added to each well of a sterile 96-well microplate (Thermo Scientific™ Sterilin™, Waltham, MA, USA). Plates were incubated at 35 °C aerobically for 24–48 h, and minimum inhibitory concentration values (MICs) were recorded as OD at 490 nm using a microplate reader (Gen5 Microplate Reader, BioTek Instruments, Winooski, VT, USA).

### 2.3. Western Blot Analysis

Briefly, for Western blot analysis, 30 μg of proteins were loaded into a 12% polyacrylamide gel MiniPROTEAN^®^ TGXTM (Bio-Rad, Milan, Italy) followed by electrotransfer to nitrocellulose membrane TransBlot^®^ TurboTM (Bio-Rad, Milan, Italy) using TransBlot^®^ SE Semi-Dry Transfer Cell (BIO-RAD, Milan, Italy) [17,18]. Subsequently, membrane was blocked in Odyssey blocking buffer (Licor, Milan, Italy) for 1 h at room temperature. After blocking, membrane was washed three times in phosphate-buffered saline (PBS) for 5 min and incubated over-night at 4 °C with primary antibodies against CHIT1 (1:500) (anti-rabbit, Santa Cruz, Dallas, TX, USA). Membranes were then washed three times in PBS for 5 min and incubated with infrared anti-mouse IRDye800CW (1:5000) and anti-rabbit IRDye700CW secondary antibodies (1:5000) in PBS/0.5% Tween-20 for 1 h at room temperature. All antibodies were diluted in Odyssey blocking buffer. The blots were visualized using Odyssey infrared imaging scanner (Licor, Milan, Italy) and protein levels were quantified by densitometric analysis [19,20].

### 2.4. Chitotriosidase Activity Assay

Chitotriosidase enzymatic activity was determined by a fluorimetric method using 22 μM 4-methylumbelliferyl β-d-*N*,*N*′,*N*″-triacetylchitotriosidase (Sigma-Aldrich S.r.l. Milan, Italy) in citrate-phosphate buffer, pH 5.2, as previously described [21,22]. Fluorescence was read at 450 nm with a Perkin Elmer Victor3 fluorimeter (excitation wavelength 365 nm, Perkin Elmer, Milan, Italy). As control, cell-free supernatants of monocytes cultured for 24 h were used. Enzymatic activity was measured as nanomoles of substrate hydrolyzed per mL per hour (nmol/mL/h).

### 2.5. Cell Cultures

U266, MM1, SH-SY5Y, HS5, and HL60 cells were obtained from American Type Culture Collection (Manassas, VA, USA). Cell lines were maintained in RPMI medium containing 2 mM l-glutamine, supplemented with 10% fetal bovine serum (FBS) and with 100 U/mL penicillin and 100 μg/mL streptomycin at a humidified 37 °C incubator providing 5% CO_2_. Human lymphocytes and monocytes were isolated, after informed consent, from fresh buffy coat of healthy volunteers provided by the Transfusional Center of E. Muscatello Hospital—Augusta (SR). Monocytes and lymphocytes then were purified from the lymphomonocytic population by positive isolation using magnetic beads coated with goat anti-mouse CD14^+^ IgG and anti-mouse CD3^+^ IgG respectively (MiltenyiBiotec GmbH, Bergisch Gladbach, Germany) [23].

### 2.6. Cell Viability Assay

Briefly, cells were seeded in 96-well plates at 1 × 10^3^ cells/well. After 24 h of treatment with FSM 20 μL of MTT solution (5 mg/mL) was added to each well. After 3 h of incubation, formazan crystals were dissolved in 150 μL of 0.1 M HCl in isopropanol. Color intensity was measured at 570 nm with ELISA plate reader (THERMO Scientific Multiskan FC, Waltham, MA, USA).

### 2.7. Bacterial Grown Inibition Calculation

The percentage of bacterial grown inhibition for each strain was calculated according to following formula:

(1) $$\%\;\mathrm{of}\;\mathrm{inhibition}=100-\frac{\mathrm{Sample}\;Y\max\;\mathrm{at}\;16.50\;\mu g/\mu L}{\mathrm{Sample}\;Y\max\;\mathrm{positive}\;\mathrm{control}\times 100},$$

*Y*max correspond to the maximum value of CFU/mL obtained for each bacterial strain tested (alone or in combination with fish mucous). In order to calculate this parameter, all bacterial growth curves were analyzed with Gompertz function by Prism GraphPad Software 6 (GraphPad Software, California, CA, USA) (Table 1) [24].

### 2.8. Statistical Analysis

All experimental data are expressed as mean ± standard error (SD). Significance was assessed by ANOVA or Student’s *t*-test. *p* < 0.05 was considered to be statistically significant. Gompertz function was verified by *R* squared values >0.99 for all the data tested (data not shown).

## 3. Results

### 3.1. Bacterial and Fungal Growth Inhibition Curves

**Figure 1:**
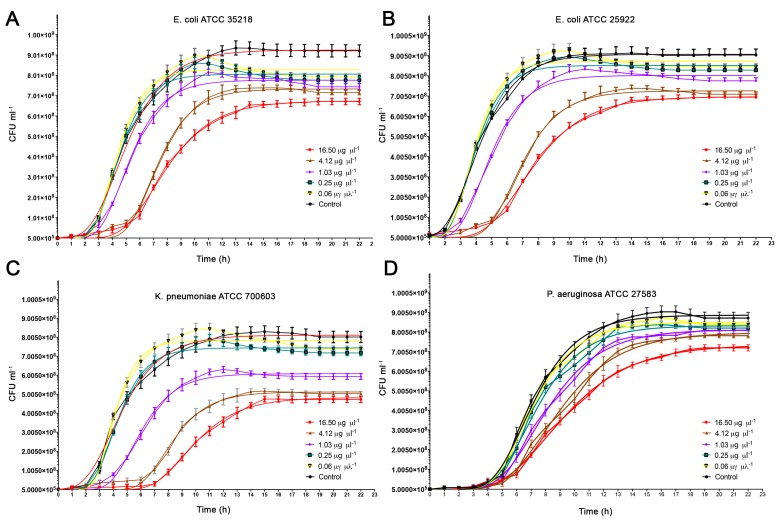
Growth curves of 4 strains (*E. coli* ATCC 35218 (**A**), *Escherichia coli* ATCC 25922 (**B**), *Klebsiella pneumoniae* ATCC 700603 (**C**), *Pseudomonas aeruginosa* ATCC 27853 (**D**)) incubated at 37 °C with fish mucous in a concentration range from 16.50 µg·µL^−1^ to 0.06 µg·µL^−1^. All experimental data are expressed as mean ± standard error (SD). Significance was assessed by ANOVA or Student’s *t*-test: *p* < 0.05.

The growth rates of the given strains compared to that of control without FSM are shown in Figure 1. Our data suggest that FSM inhibited growth of Gram-negative strains (*p* < 0.05).

A concentration of 16.50 μg/μL^−1^ reduced the growth curve of *Klebsiella pneumoniae* ATCC 700603 by 40.44%, *Escherichia coli* ATCC 35218 by 27.05%, *E. coli* ATCC 25922 by 23.15%, and *Pseudomonas aeruginosa* ATCC 27853 by 17.02%. Interestingly, FSM did not show any growth curve inhibition of tested Gram-positive bacteria (i.e., *Enterococcus faecalis* ATCC 29212, *Staphylococcus aureus* ATCC 25923, *Streptococcus agalactiae* DSM 2134). The latter finding was probably due to the presence of outer membrane in Gram-negative strains that could have been damaged by FSM leading to a growth reduction or more likely by agglutination as suggested by Guan et al. [25].

Antifungal activity defined as MIC values, i.e., the prominent decrease in turbidity corresponding to approximately 50% inhibition of growth was determined spectrophotometrically [3]. The results carried out MIC values are indicated in Table 2. All the strains were inhibited at 4.12 µg/µL and no differences were observed in the susceptibilities of both standard and clinical isolated strains (Table 2).

### 3.2. Chitinase Expression and Activity

**Figure 2:**
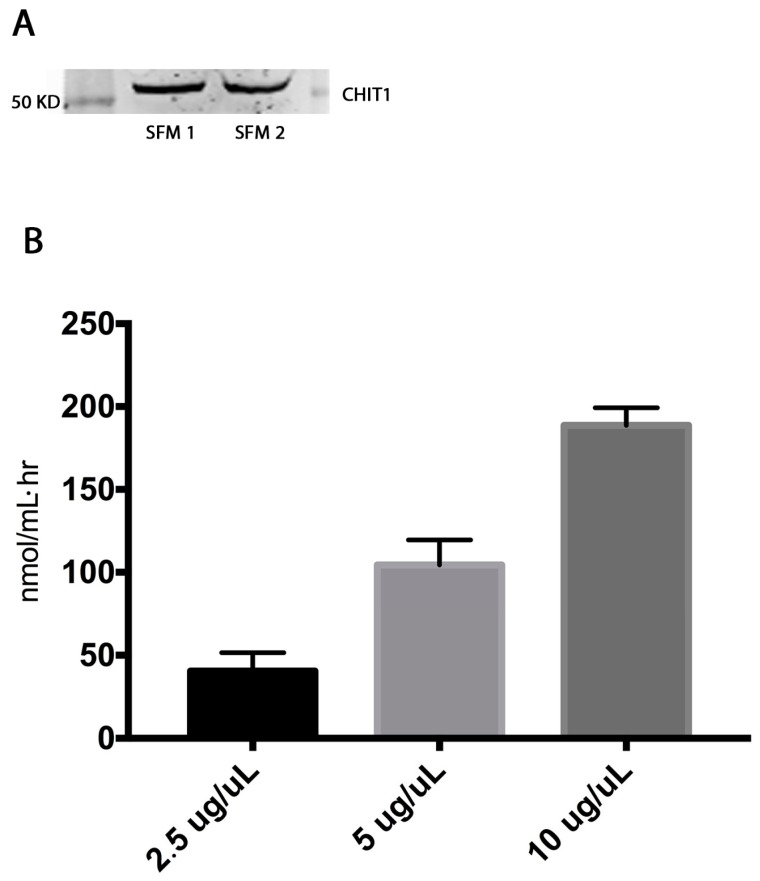
(**A**) Western blot assay showed the presence of CHIT1 protein in FSM. (**B**) Enzyme activity was performed by fluorimetric assay and was expressed as nanomoles per mL per hour in FSM. Data are expressed as mean ± SD of at least three independent experiments.

Western blot analysis showed CHIT1 expression (55 KD) in FSM of *Dasyatis pastinaca*. In addition, the enzymatic activity of CHIT1 was measured; interestingly, (Figure 2) we observed a significant (*p* < 0.05) increase of enzymatic activity in a dose dependent manner (2.5 µg/µL: about 50 nmol/mL·h, 5 µg/µL: about 100 nmol/mL·h; 10 µg/µL: about 200 nmol/mL·h).

### 3.3. Anti-Proliferative Activity

**Figure 3:**
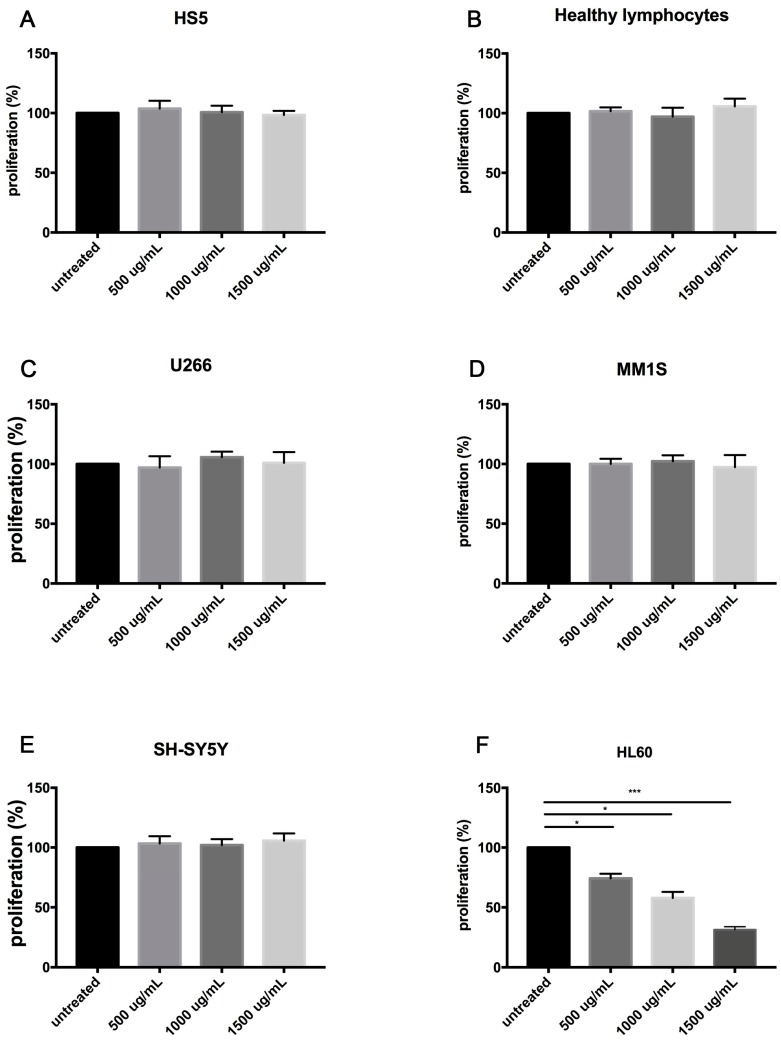
MTT assay in (**A**) HS5 cells normal bone marrow/stroma cells, (**B**) healthy lymphocytes, (**C**) U266 multiple myeloma cell lines, (**D**) MM1S multiple myeloma cell lines, (**E**) SH-SY5Y neuroblastoma cell lines, (**F**) HL60 acute promyelocytic leukemia cell lines, after treatment with 500 µg/mL, 1000 µg/mL, and 1500 µg/mL of FSM. Bars represent the mean ± SEM of four independent experiments. *** *p* < 0.0001, * *p* < 0.01, versus untreated cells.

The anti-proliferative activity of the FSM was tested in U266, MM1, SH-SY5Y, HL60 cancer cell lines and in healthy cells such as SH5 lymphocytes cells, at different concentrations (500 µg/mL; 1000 µg/mL and 1500 µg/mL of FSM). After 24 h of treatment, we observed the inhibition of cell proliferation in a dose-dependent manner (*p* < 0.0001) (Figure 3) only in HL60 cell lines (*p* < 0.001). The U266, MM1, and SH-SY5Y cells showed no sensitivity to FSM treatment (Figure 3). Furthermore, in healthy lymphocytes and mesenchymal stem cells, FSM treatment had no significant effects (Figure 3).

## 4. Discussion

Fish skin or mucus may represent a good source of biologically active compounds to be used for different medical purposes. The biological interface between fish and their aqueous environment consists of a mucus layer composed of biochemically diverse secretions from epidermal and epithelial cells. Interestingly, many anti-microbial peptides are active versus both Gram-positive and Gram-negative bacteria but few bactericidal peptides have been shown to exhibit anticancer and antiviral properties [26,27]. Consistently with these observations, several lines of evidence suggest that the occurrence of infections in fish is very rare [26,28], thus leading scientists to study fish biological interface. Our data suggest that FSM is able to inhibit microbial growth against Gram-negative bacteria, but not against Gram-positive bacteria. In particular, among Gram-negative strains, *Klebsiella pneumoniae* was the most sensitive strain, followed by two strains of *Escherichia coli* (respectively reduction of 27.05% and 23.15%) and *Pseudomonas aeruginosa* (only 17.02% of growth inhibition). This selectivity may be explained by the nature of the active molecules present in the FCM. In fact, most antimicrobial peptides should possess two requirements, the net cationic charge and the ability to take amphipathic structures. The first property allows the attraction of bacterial membranes having negative charge, especially Gram-negative ones, and the second property allows the interaction and absorption inside bacteria [29]. FCM could have a greater effect on Gram-negative strains rather than Gram-positive ones because of a strong interaction between the outer membrane (present only in the Gram-negative strains) and the active molecules present in the FCM. Furthermore, Guan et al. [24] suggested that this interaction could cause damage by growth reduction or more likely by agglutination. As far as concern the antifungal activity, MIC values showed a great inhibition against four strains of *Candida albicans*, *Candida glabrata*, and *Candida tropicalis*. Such results are consistent with the presence of chitinase found in FSM, which may be responsible for the observed strong antifungal activity [30]. However, it should be taken into due account that FSM contains several active compounds, which may be responsible for the observed biological effects. In particular, previous authors showed that *Larimichthys crocea* contains more than 3000 proteins in skin mucus and 521 proteins were also found in salmon mucus [31,32]. In this regard, mucus proteins may derive from several routes including that from dead cells and this may explain, at least in part, the large numbers of proteins present in the mucus. Several studies have examined the differential proteome profile in fish after immune relevant challenges such as infection, stress, and probiotic exposure [31,32,33]. Furthermore, FSM contains well-established antimicrobial proteins such as lysozyme, a different type of lectin, several types of heat shock proteins, apolipoprotein 1, and complement [34,35,36,37]. We demonstrated in this study that CHIT1 protein is also present in FSM of *Dasyatis pastinaca* and it showed a high enzymatic activity. The family of chitinases includes members both with and without glycohydrolase enzymatic activity against chitin. CHIT1 and acidic mammalian chitinase (CHIA or AMCAse) are the only two true chitinase possessing chitinolytic (glycohydrolase) activities [38]. Elevated levels of chitinases have been reported in a variety of diseases including infections, chronic inflammation, and degenerative disorders [39,40]. Recent studies reported that CHIT1 play a role as antifungal and antibacterial activity [41,42,43,44,45]. We also demonstrated an enzymatic activity of CHIT1 in FSM that can partially explain its anti-microbial activity.

## 5. Conclusions

Our results suggest that FSM exhibit significant and specific antimicrobial and antifungal activities and possess cytotoxic effects against specific cancer cell lines in the absence of evident toxic effects on non-tumoral cells. Therefore, this work, offers insight on the development and identification of chemotherapic agents to overcome microbial and tumoral chemoresistance.

Future studies are now warranted in order to identify specific compounds in FSM responsible for the observed effects in order to provide new pharmacological strategies for antimicrobial and anti tumoral activity.

## Figures and Tables

**Table 1:** Statistical analysis and growth inhibition percentage in presence of the highest concentration tested of crude fish mucous.

Strains Tested	*Escherichia coli* ATCC 35218		*Escherichia coli* ATCC 25922		*Klebsiella pneumoniae* ATCC 700603		*Pseudomonas aeruginosa* ATCC 27853	
	16.50 µg/µL (22 h)	Control+ (22 h)	16.50 µg/µL (22 h)	Control+ (22 h)	16.50 µg/µL (22 h)	Control+ (22 h)	16.50 µg/µL (22 h)	Control+ (22 h)
Ymax (CFU/mL)	6.74 × 10^8^	9.24 × 10^8^	6.97 × 10^8^	9.07 × 10^8^	4.83 × 10^8^	8.11 × 10^8^	7.36 × 10^8^	8.87 × 10^8^
Ymax Standard Error (CFU/mL)	4.99 × 10^6^	6.08 × 10^6^	5.39 × 10^6^	4.17 × 10^6^	3.23 × 10^6^	7.37 × 10^6^	5.91 × 10^6^	7.36 × 10^6^
Inhibition (%)	27.05		23.15		40.44		17.02	

Control+: positive control.

**Table 2:** Microdilution assay of crude fish mucous for antifungal activity: Minimum inhibitory concentration values (MICs) were recorded as OD at 490 nm after 24 h and 48 h for each strain.

Strains	MIC µg·µL^−1^	
	24 h	48 h
*Candida albicans* ATCC90028	4.12	4.12
*Candida albicans* ATCC10231	4.12	4.12
*Candida albicans* (6) clinical strain	4.12	4.12
*Candida albicans* (10) clinical strain	4.12	4.12
*Candida glabrata* (14) clinical strain	4.12	4.12
*Candida tropicalis* (21) clinical strain	4.12	4.12

Number in brackets is the reference number of the clinical isolate.
